# cAMP-Dependent Signaling Restores AP Firing in Dormant SA Node Cells via Enhancement of Surface Membrane Currents and Calcium Coupling

**DOI:** 10.3389/fphys.2021.596832

**Published:** 2021-04-09

**Authors:** Kenta Tsutsui, Maria Cristina Florio, Annie Yang, Ashley N. Wirth, Dongmei Yang, Mary S. Kim, Bruce D. Ziman, Rostislav Bychkov, Oliver J. Monfredi, Victor A. Maltsev, Edward G. Lakatta

**Affiliations:** ^1^Laboratory of Cardiovascular Science, Biomedical Research Center, Intramural Research Program, National Institute on Aging, NIH, Baltimore, MD, United States; ^2^Department of Cardiovascular Medicine, Faculty of Medicine, Saitama Medical University International Medical Center, Saitama, Japan; ^3^Heart and Vascular Center, University of Virginia, Charlottesville, VA, United States

**Keywords:** cardiac automaticity, pacemaker mechanism, dormant cells, coupled-oscillator system, sinoatrial nodal cells

## Abstract

Action potential (AP) firing rate and rhythm of sinoatrial nodal cells (SANC) are controlled by synergy between intracellular rhythmic local Ca^2+^ releases (LCRs) (“Ca^2+^ clock”) and sarcolemmal electrogenic mechanisms (“membrane clock”). However, some SANC do not fire APs (dormant SANC). Prior studies have shown that β-adrenoceptor stimulation can restore AP firing in these cells. Here we tested whether this relates to improvement of synchronization of clock coupling. We characterized membrane potential, ion currents, Ca^2+^ dynamics, and phospholamban (PLB) phosphorylation, regulating Ca^2+^ pump in enzymatically isolated single guinea pig SANC prior to, during, and following β-adrenoceptor stimulation (isoproterenol) or application of cell-permeant cAMP (CPT-cAMP). Phosphorylation of PLB (Serine 16) was quantified in the same cells following Ca^2+^ measurement. In dormant SANC LCRs were small and disorganized at baseline, membrane potential was depolarized (−38 ± 1 mV, *n* = 46), and I_CaL_, I_f_, and I_K_ densities were smaller vs SANC firing APs. β-adrenoceptor stimulation or application of CPT-cAMP led to *de novo* spontaneous AP generation in 44 and 46% of dormant SANC, respectively. The initial response was an increase in size, rhythmicity and synchronization of LCRs, paralleled with membrane hyperpolarization and small amplitude APs (rate ∼1 Hz). During the transition to steady-state AP firing, LCR size further increased, while LCR period shortened. LCRs became more synchronized resulting in the growth of an ensemble LCR signal peaked in late diastole, culminating in AP ignition; the rate of diastolic depolarization, AP amplitude, and AP firing rate increased. I_CaL_, I_K_, and I_f_ amplitudes in dormant SANC increased in response to β-adrenoceptor stimulation. During washout, all changes reversed in order. Total PLB was higher, but the ratio of phosphorylated PLB (Serine 16) to total PLB was lower in dormant SANC. β-adrenoceptor stimulation increased this ratio in AP-firing cells. Thus, transition of dormant SANC to AP firing is linked to the increased functional coupling of membrane and Ca^2+^ clock proteins. The transition occurs via (i) an increase in cAMP-mediated phosphorylation of PLB accelerating Ca^2+^ pumping, (ii) increased spatiotemporal LCR synchronization, yielding a larger diastolic LCR ensemble signal resulting in an earlier increase in diastolic I_NCX_; and (iii) increased current densities of I_f_, I_CaL_, and I_K_.

## Introduction

Spontaneous electrical impulses that drive the heartbeat originate in the sinoatrial node (SAN). Early studies have discovered that automaticity of SAN pacemaker cells (SANC) is mainly driven by a combination of voltage-dependent activation and inactivation of ion channels (Noble, 1984). Pacemaker mechanism was interpreted initially as the time-dependent decline of K^+^ conductance, which unmasked a background inward Na^+^ current (Noble, 1962). This was followed by the discovery of the “funny current,” (DiFrancesco et al., 1986), i.e., an inward current I_f_ which is activated by membrane hyperpolarization, and was next believed to be the pacemaker current. Although this interpretation was later revealed to be oversimplified: other sarcolemmal ion channels, in addition to I_f_ and K^+^ channels, were shown to be crucial for SANC automaticity (Wilders et al., 1991; Irisawa et al., 1993; Noma, 1996; Mangoni and Nargeot, 2008; Mesirca et al., 2015). In reality, these ion channels’ functions are tightly integrated. For example, openings of low-voltage activated L-type Ca^2+^ channels Ca_v1.3_ (Mangoni and Nargeot, 2008) occur during diastolic depolarization and subsequent activation of Ca_v1.2_ channels generates an action potential (AP) upstroke. The resultant membrane depolarization activates voltage-activated K^+^ channels leading to AP repolarization. This chain reaction of ion channel activation has been referred as to “membrane clock” or “M clock” (Lakatta et al., 2010).

Recent progress in understanding mechanisms of SANC automaticity has revealed even more complexity in the pacemaker mechanisms that involve intracellular Ca^2 +^ cycling (Rubenstein and Lipsius, 1989; Rigg and Terrar, 1996; Huser et al., 2000; Bogdanov et al., 2001). Spontaneous AP generation is contributed by roughly periodic, spontaneous diastolic local Ca^2+^ releases (LCRs), generated via spontaneous activation of ryanodine receptors (RyR2) of the sarcoplasmic reticulum (SR), i.e., “Ca^2+^ clock” coupled to the M clock. Diastolic activation of the Na^+^-Ca^2+^-exchanger (NCX) by LCRs, results in larger net inward current (I_NCX_) that, in the context of actiavated I_f_ and decaying K^+^ conductance, accelerates diastolic depolarization toward the AP threshold, review (Lakatta et al., 2010). The most recent conceptualization of these complex interactions among multiple pacemaker mechanisms has been redefined as an ignition process (Lyashkov et al., 2018) that complements aforementioned signaling from LCRs and I_NCX_ to depolarize cell membrane with additional activation of low voltage activated Ca^2+^ channels (Ca_v1.3_ and Ca_v3.1_) generating diastolic Ca^2 +^ current (I_CaL_ and I_CaT_) and attendant Ca^2+^ influx to activate more LCRs via Ca^2+^-induced-Ca^2+^-release (Chen et al., 2009; Torrente et al., 2016) that, in turn, generates more I_NCX_ and membrane depolarization, forming an explosive feed-forward loop to insure the attainment of AP threshold and generation of a new pacemaker cycle.

The robust biophysical “engine” of automaticity described above is, in turn, driven and regulated by a biochemical “engine”: Ca^2+^-calmodulin-activated adenylyl cyclase in SANC increases cAMP (Mattick et al., 2007; Younes et al., 2008) that activates I_f_ [by shifting its activation curve (DiFrancesco and Tortora, 1991)] and cAMP-mediated PKA-dependent and CaMKII-dependent phosphorylation of clocks’ proteins resulting in increased Ca^2 +^ releases that feed-forward activities of the proteins (L-type Ca^2 +^ channels, RyR, PLB (phospholamban), and SERCA). The activation level of the biochemical “engine” under basal conditions is kept near a mid-range by phosphatases and phosphodiesterases (Vinogradova et al., 2008; Lakatta et al., 2010).

Marked variation exists in AP firing intervals among individual isolated SANC (Opthof et al., 1987; Lyashkov et al., 2007; Yaniv et al., 2014). In addition to this cell-to-cell variability of average AP intervals at baseline (inter-SANC variability), beat-to-beat variability in the AP cycle intervals also occurs in each SANC (*intra*-SANC variability) (Wilders and Jongsma, 1993). Emerging evidence suggests that the degree of clock synchronization (i.e., functional coupling between the Ca^2+^- and M clocks) fluctuates on a beat-to-beat basis, and that this is the basis of intra-SANC variability (Monfredi et al., 2013), with the more effective coupling associated with higher rates and smaller variability (Yaniv et al., 2014). Thus, we define clock coupling as various degrees of synchronization of multiple mechanisms both in the cell membrane and inside the cell with the AP cycle length (CL) and its cycle-to-cycle variability reporting the effectiveness of the coupling.

β-adrenoreceptor (AR) stimulation of isolated SANC enhances clock synchronization and coupling in part via both direct cAMP effects and also by phosphorylation of multiple clock proteins to increase the AP firing rate (Lakatta et al., 2010) and reduce the variability of AP firing intervals both within and among isolated SANC (Yaniv et al., 2016; Kim et al., 2018; Yang et al., 2020), while cholinergic receptor stimulation has the opposite effect (Lyashkov et al., 2009). Thus, flexible degrees of effectiveness of clock coupling deviating from its mid-range in SANC determine both the average AP firing intervals and AP firing interval variability harboring the entire physiologic range of steady-state AP firing.

Recent studies of single isolated SANC revealed that cells that do not fire APs (dormant cells) still generate LCRs, and a large proportion of these cells began to fire spontaneous AP-induced Ca^2+^ transients in response to β-AR stimulation (Kim et al., 2018). However, APs, ion currents, and protein phosphorylation were not measured in that study. Here we propose that (i) dormant cells have insufficient clock coupling to fire APs, i.e., their clock coupling extremely deviates from the aforementioned mid-range regulation, even beyond what cholinergic receptor stimulation does in AP firing cells; (ii) in transition to AP firing β-AR stimulation increases key ion currents, phosphorylation, and membrane potential oscillations, in addition to Ca^2 +^ signaling to make clock protein functions more synchronized, i.e., enhancing clock coupling.

## Materials and Methods

### SANC Isolation and Selection

All animal studies followed the Guide for the Care and Use of Laboratory Animals published by the National Institutes of Health (NIH Publication no. 85-23, revised 1996). Experimental protocols were approved by the Animal Care and Use Committee of the National Institutes of Health (protocol #034LCS2016). SANC were isolated from guinea pig (Kim et al., 2018). A beating cell was defined as a SANC with apparent spontaneous contractions. On the other hand, a non-beating cell was a SANC lacking contractions. Only SANC with “spindle” “spider” or “rod” appearance (Denyer and Brown, 1990; Verheijck et al., 1998; Boyett et al., 2000) were chosen. Apparently damaged cells on the basis of structure were excluded from this study.

### Electrophysiology

All electrophysiological signals, APs and ion currents, were measured by a patch-clamp amplifier Axopatch 200B, digitized with DIGIDATA 1440A, and recorded (on-line) and analyzed (off-line) with pClamp software version 10 (all from Molecular devices, PA, United States).

A perforated patch clamp method was used to measure membrane potentials (Rigg et al., 2000). Briefly, isolated SANC fixed in a heated bath (36 ± 0.5°C) were superfused at a rate of 1 ml/min with the HEPES-based solution of which temperature was kept at 36 ± 0.5°C and consisted of the following composition: 140 NaCl; 5 KCl; 5 HEPES; 0.33 NaH_2_PO_4_; 5.5 Glucose; 0.5 MgCl_2_; 1.8 CaCl_2_, titrated to pH 7.3 with NaOH. Glass micro-pipettes (resistance 3–5 MΩ) were filled to closely emulate intracellular composition (mM): 143 KCl; 10 NaCl; 2 Mg ATP; 5 HEPES; 10 EGTA; pH 7.3 adjusted with KOH. Amphotericin B (250 μM) was used to measure the membrane potential of SANC. The continuous recording was undertaken before, during and after application of either β-AR agonist isoproterenol (100 nM) or cell-permeable CPT-cAMP (300 μM).

To compare the ion current density profile of dormant SANC with that of firing SANC, a whole-cell patch clamp method was used as previously described (Monfredi et al., 2018). In short, the voltage protocols and pipette solution were designed to measure major ionic currents consecutively in the same cell (always in the following order: I_CaL_, I_f_, and I_K_). The patch pipettes had resistances ranging between 2 and 3 MΩ, and were filled with the following solution (in mM): K^+^ gluconate 100, MgATP 2.5, Na_2_ATP, HEPES 5, KCl 20, EGTA 5, CaCl_2_ 2; titrated to pH 7.2 with KOH. Tetrodotoxin (10 μM) was added to the bathing solution to block Na^+^ currents that could otherwise interfere with I_CaL_ measurements. The cell capacitance and series resistance were electronically compensated by the amplifier to the point just preceding positive feedback oscillations. Seal resistance was measured at the beginning of each experiment and was routinely >10 GΩ. If the seal resistance was lower than this, data from the cell were discarded. Capacitance currents were measured by applying a ramp from −60 to −80 mV. We used a ramp with a 10 V/s rate of change of membrane potential (20 mV over 2 ms) and measured resultant current at the end of the ramp, which we used to assess electrical membrane capacitance. Measured ionic currents were normalized to cell capacitance, to yield a current density in pA/pF (Monfredi et al., 2018).

#### I_CaL_ Measurements

In line with previous studies (Honjo et al., 1996), depolarizing steps lasting 300 ms from a holding potential of −45 mV were undertaken, with a first level of −40 mV, in 5 mV increments, to a final level of +40 mV. IV curves were plotted in each cell, and the I_CaL_ current density was taken to be the peak current at 0 mV.

#### I_f_ Measurements

In line with previous studies (Honjo et al., 1996), hyperpolarizing steps lasting 1000 ms from a holding potential of −35 mV were undertaken, with a first level of −40 mV, in −10 mV increments, to a final level of −120 mV. IV curves were plotted in each cell, and the I_f_ current density was taken to be the mean current at −110 mV over a 50 ms period beginning 300 ms after the hyperpolarizing pulse. This protocol has a short, 300 ms pulse duration that does not fully saturate I_f_ activation, and aims to quickly evaluate effective I_f_ magnitude during diastole, rather than its maximum conductance, activation curve, and kinetics. Thus, the I_f_ magnitude increase in our experiments with β-AR stimulation reflects both the shift of the activation curve to more positive potentials and acceleration of activation kinetics.

#### I_K_ Measurements

In line with previous studies (Lei and Boyett, 1999; Lei et al., 2000), depolarizing steps lasting 1000 ms from a holding potential of −60 mV were undertaken, with a first level of −60 mV, in +10 mV increments, to a final level of 50 mV. IV curves were plotted in each cell, and the I_K_ current density was taken to be the mean current at +40 mV over a 50 ms period beginning 300 ms after the hyperpolarizing pulse.

### 2D Intracellular Ca^2+^ Signal Measurement

Cells were loaded with 5 μM Fluo-4AM for 20 min at a room temperature before the measurement. During measurement, cells were continuously perfused with HEPES-based saline at 36 ± 0.1°C by temperature controller TC2BIP 2/3Ch (Cell MicroControls, Norfolk, VA, United States). Ca^2+^ signals were imaged with a 2D camera sCMOS PCO edge 4.2 with a 13.2 mm square sensor of 2048 × 2048 pixels resolution. To resolve LCR dynamics, we acquired images at a rate of 100 frames/second that was possible only using a part of the sensor (1280 × 1280). The recording camera was mounted on Zeiss Axiovert 100 inverted microscopes (Carl Zeiss, Inc., Germany) with a ×63 oil immersion lens and a fluorescence excitation light source CoolLED pE-300-W (CoolLED Ltd., Andover, United Kingdom). Fluo-4 fluorescence excitation (470/40 nm) and emission light collection (525/50 nm) were performed using the Zeiss filter set 38 HE. When the Ca^2+^ signals were simultaneously measured with membrane potential, we programmed the patch clamp amplifier to timely shoot out a short TTL signal, wired to the 2D camera that was also set to start recording upon receiving the synchronizing pulse from patch amplifier. The 2D video-recording of intracellular Ca^2+^ dynamics was exported and analyzed by a computer program that detects LCRs and quantifies the size, period (a time interval between the peak of the prior AP-induced Ca^2+^ transient and the onset of an LCR), and the ensemble area of LCRs (the sum area of all detected LCR signals) as previously described (Maltsev et al., 2017). Our previous studies using confocal microscopy in the line-scanning mode demonstrated that β-AR stimulation indeed increases number of LCRs (Figure 4A in Vinogradova et al., 2002). However, in the present study, using 2D non-confocal imaging, we measured and LCR ensemble area. We report this parameter because the total area of all LCRs is what activates NCX current and contributes to the diastolic depolarization.

### Dual Immunostaining of Phospholamban

Immediately after Ca^2 +^ measurements up to 1 h, the cells were fixed for 10 min at room temperature with 4% formaldehyde in phosphate buffer (PBS), and the samples were stored in 0.1% Triton/PBS at 4°C for subsequent immunostaining and confocal imaging.

Dishes were washed twice with washing solution (0.1% Triton/PBS) and permeabilized with 0.5% Triton-X-100 in PBS for 15 min at room temperature. The samples were again washed twice then blocked by incubating overnight in a solution containing: 2% BSA/PBS, 5% normal goat serum, 0.02% NaN3, and 0.1% Triton. SANC were then incubated with anti-PLB total antibody (mouse, 1:200, Badrilla, Cat. No. A010-14) and anti-phosphorylated phospholamban (PLB) at Ser 16 antibody (rabbit, 1:200, Badrilla, Cat. No. A010-12AP) at 4°C overnight. Cell were then washed 5–10 min followed by labeling with Atto647 goat anti-mouse IgG (1:1000, Sigma, Cat. No. 50185) and Cy3 goat anti-rabbit IgG secondary antibody (1:1000, Jackson ImmunoResearch, Cat. No. 111-165-144) followed by additional incubation for 1 h at room temperature, and finally washed with plain PBS for 5 min. Plates were mounted with ProLong Gold antifade reagent (Thermo Fisher Scientific, Cat. No. P36934).

### Confocal Imaging

Fluorescence was imaged by a Zeiss LSM 880 confocal microscope (Carl Zeiss Inc., Germany) using a 40×/1.3 N.A. oil immersion lens. The Atto647 and Cy3 fluorophores were excited with 633 nm (DPSS 10 mW) and 561 nm (He-Ne 5 mW) lasers, respectively. Individual SANC captured in live Ca^2+^ imaging was identified by its grid location and cell morphology. Images were processed and the intensity of immunofluorescent signal was quantified using ZEN 2.3 lite software (Carl Zeiss Inc., Germany). The fluorescence density of phosphorylated phospholamban was normalized to that of the total phospholamban.

### Preparation for Live Intracellular Imaging

Laminin (Sigma-Aldrich, St. Louis, MO, United States; Cat. No. L2020) – coated dishes and physiological bathing solution (Tyrode, or HEPES-based saline) were prepared fresh the day of each experiment. Glass bottom gridded dishes (MatTek Corporation, Ashland, MA, United States; Cat. No. P35G-1.5-14-CGRD) were coated with 40 μg laminin/mL PBS + 1.0% PS in the center of the coverslip and incubated for 1 h before aspirating.

### Statistical Analysis

Data are presented as mean ± SEM. *p* < 0.05 was considered statistical significance. Unpaired, two-tailed *t*-test was used to test differences in current densities and AP characteristics between (i) dormant and firing SANC in baseline and (ii) DMSO- and ivabradine (IVA)-pretreated responder dormant SANC. One-way ANOVA with Tukey’s multiple comparisons test was used to compare current densities and AP characteristics. Fisher’s exact test was used to compare the response% of cyclopiazonic acid (CPA)- or IVA-treated dormant SANC with that of DMSO-pretreated dormant SANC.

## Results

### Characterization of Membrane Potential, Ion Current Density Profile, and LCRs in Dormant SANC at Baseline

**FIGURE 1:**
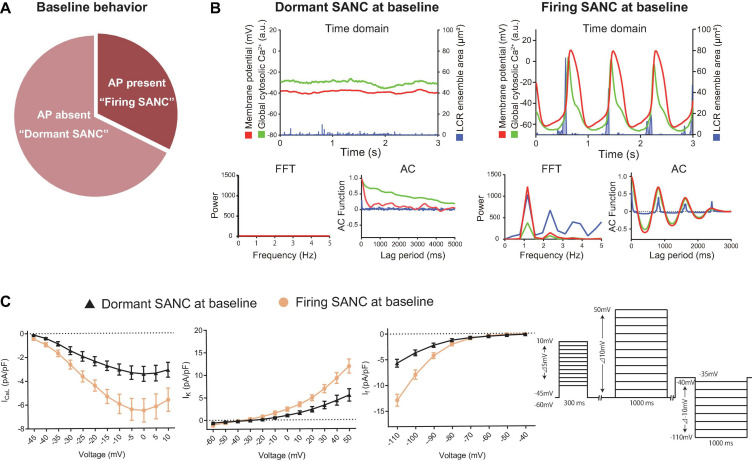
**(A)** The proportion of baseline behavior of freshly isolated guinea pig SANC we studied total 34 cells. About 1/3 and 2/3 were AP present (firing) and AP absent (dormant), respectively. **(B)** Illustrative examples of simultaneous voltage (magenta), global cytosolic Ca^2+^ (green) and LCR ensemble area (blue) in dormant SANC (left) and firing SANC (right) at baseline. The corresponding frequency analysis by fast Fourier transform (FFT) and autocorrelation analysis (AC) are shown below. **(C)** Major ion current density of voltage-dependent L-type Ca^2+^ channel, voltage-dependent K channel and the funny current in dormant (black) and firing (yellow) SANC at baseline; those of dormant SANC were reduced compared to those of firing SANC. The protocol is shown in the right panel.

We surveyed AP firing behavior of 34 freshly isolated single SANC from 10 guinea pig hearts. Of the 34 SANC, 11 manifested spontaneous AP firing at baseline (“firing SANC”) while 23 did not (“dormant”) (Figure 1A). In contrast to SANC that fired spontaneous APs at baseline, in which the maximum diastolic potential (MDP) was −58.5 ± 1.2 mV (Table 1), the membrane potential of dormant SANC was more depolarized to −39 ± 0.2 mV (Figure 1B top left and Table 1).

**TABLE 1:** Membrane potential characteristics of initially firing/dormant guinea pig SANCs.

	**AP Firing SANC**		**Dormant SANC**				
			**Overall**	**Responder**		**Non-responder**	
	**Baseline**	**β-AR stimulation**	**Baseline**	**Baseline**	**β-AR stimulation**	**Baseline**	**β-AR stimulation**
*n*	7	7	12	6	6	6	6
CL (ms)	892 ± 124	484 ± 39*	NA	NA	509 ± 35*	NA	NA
CV (%)	13.3 ± 3.2	5.5 ± 1.1	NA	NA	5.5 ± 1.2	NA	NA
MDP (mV)	−58.5 ± 1.2	−66.5 ± 0.8*	NA	NA	64.0 ± 1.9*	NA	NA
Dormant potential (mV)	NA	NA	−39.5 ± 0.9^#^	−40.1 ± 1.5	NA	−39.0 ± 1.2	−39.9 ± 1.3
AP amplitude (mV)	72 ± 3	89 ± 4*	NA	NA	76 ± 6	NA	NA
Ignition period (ms)	802 ± 118	402 ± 46*	NA	NA	426 ± 42*	NA	NA

CL, cycle length; MDP, maximum diastolic potential; NA, not applicable. *p < 0.05 vs those of firing at baseline control by one-way ANOVA with Tukey multiple comparisons. ^#^*p* < 0.05 vs MDP of firing at baseline control by unpaired *t*-test. CV stands for coefficient of variance (calculated as standard deviation divided by mean).

To determine the baseline functional profile of the M clock in dormant and firing SANC, we sequentially measured current density profiles of the L-type Ca^2+^ current (I_CaL_), funny current (I_f_), and summed repolarizing K^+^ currents (I_K_) in another 7 firing and 15 dormant cells, as previously described (Monfredi et al., 2018). Current-voltage (I–V) relationships of all three currents (Figure 1C) reflect a reduction in all three currents in dormant SANC vs those firing spontaneous APs at baseline (Figure 1C, *p* < 0.05 indicated as ^∗^).

To characterize baseline intracellular Ca^2+^ signaling of dormant and spontaneously firing SANC, we loaded other 14 firing SANC and 45 dormant SANC with Fluo-4, a fluorescent Ca^2+^ indicator. Consistent with our previous study (Kim et al., 2018), all SANC studied, both dormant and those firing spontaneous APs, exhibited LCRs at baseline (Figure 1B top). Fast Fourier transform (FFT) and autocorrelation analyses in SANC firing APs at baseline showed robust rhythmicity in membrane potential, global cytosolic Ca^2+^ signal, and LCR ensemble area, and *vice versa* confirmed a lack of spontaneous rhythmicity of these parameters in dormant SANC (Figure 1B bottom). Thus, while firing SANC produce rhythmic ensemble LCR signals synchronized to late diastole, LCRs of dormant SANC were smaller and less organized.

**FIGURE 2:**
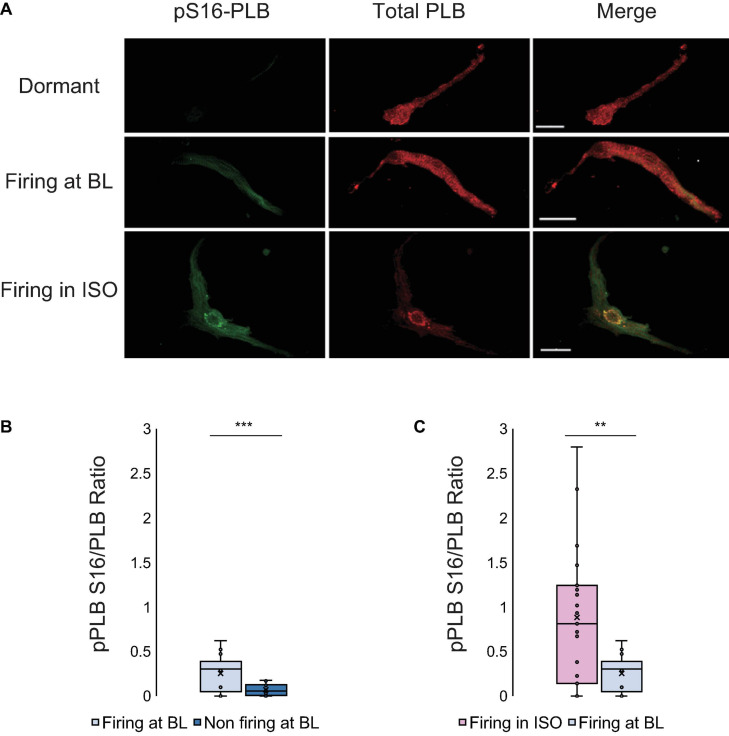
**(A)** Double immunolabeling of phosphorylated phospholamban (p-PLB) at Serine 16 (green) and total PLB (red) and the merged images of SANC that were dormant at baseline (top), firing at baseline (middle) and dormant at baseline but began firing in response to isoproterenol (bottom). **(B)** The p-PLB/total PLB ratio of SANC firing at baseline (*N* = 3/*n* = 10) vs dormant at baseline (*N* = 3/*n* = 10) (At baseline, firing cells have much higher pPLB S16/PLB ratio than non-firing cells)] and **(C)** The p-PLB/total PLB ratio of SANC firing in isoproterenol (*N* = 5/*n* = 15) vs firing at baseline (*N* = 3/*n* = 10) (Firing cells in ISO have higher pPLB S16/PLB ratios than firing cells at BL). *t*-test, ***p < 0.001, **p < 0.01.

PLB phosphorylation at Serine 16 (Figure 2A), a crucial determinant of LCR kinetics and synchronization (Sirenko et al., 2012), was reduced in dormant SANC vs AP firing SANC at baseline (*p* < 0.001, Figure 2B).

### Dormant SANC Begin to Generate Spontaneous AP in Response to Enhanced cAMP-PKA Signaling

Because enhancing cAMP-PKA signaling by β-AR stimulation increases an AP firing rate in firing SANC via facilitation of the functional coupling between Ca^2+^ and M clocks (Vinogradova et al., 2002; Tsutsui et al., 2018), we sought to discover whether cAMP-PKA signaling was capable of inducing *de novo* spontaneous APs in dormant SANC at baseline.

**FIGURE 3:**
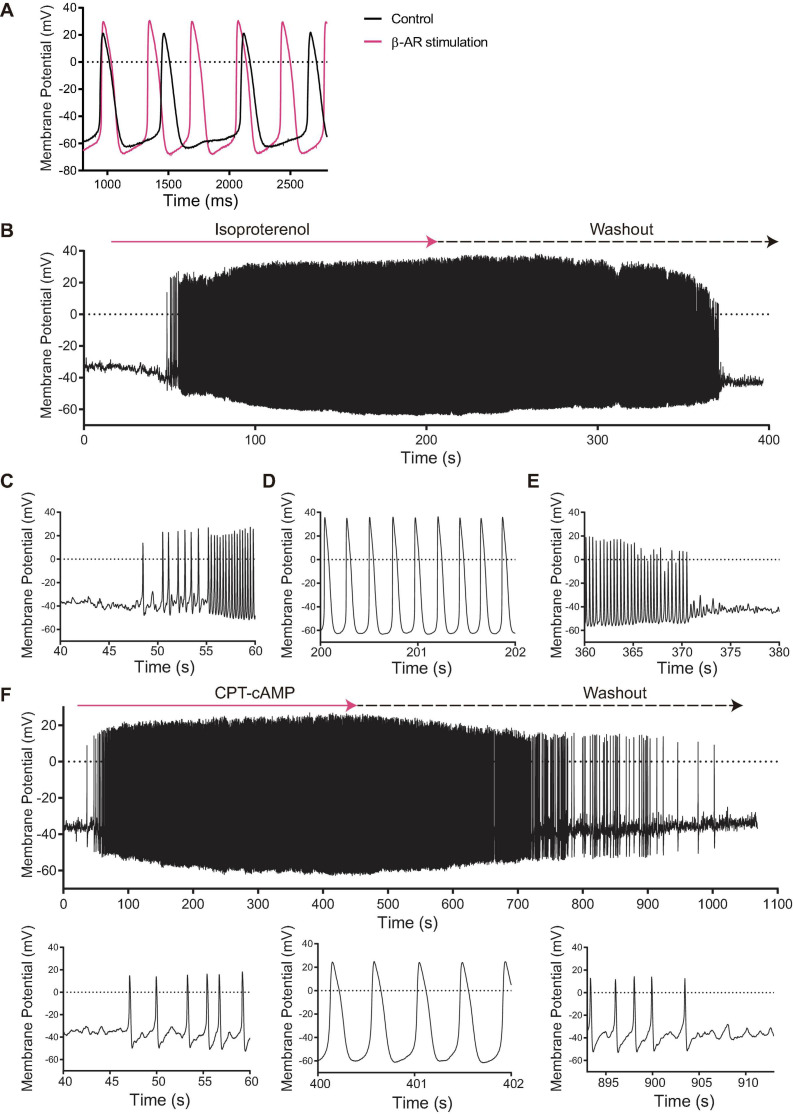
**(A)** Membrane potential tracings of SANC that fire spontaneous APs at baseline (black) that accelerated in response to isoproterenol (magenta). **(B)** Among 12 initially dormant SANC, 44% fired APs in response to β-AR stimulation (“responder”) while 56% failed to do so (“non-responder”). This was significantly different from the corresponding time control group (*n* = 8) in which spontaneous AP firing did not occur (*p* < 0.05).) When isoproterenol was washed out, the β-AR stimulation-induced APs stopped. Detail of the beginning **(C)**, steady state **(D)** and wash-out **(E)** phases of the β-AR stimulation-induced AP in a SANC that was dormant under initial baseline conditions. **(F)** The cell permeable CPT-cAMP recapitulated the β-AR stimulation-induced APs in other set of SANC that were dormant at baseline. Note the similarity to panel **(B–E)**.

β-AR stimulation with 100 nM isoproterenol of SANC firing APs at baseline reduced AP CL by 46% (892 ± 39 to 484 ± 39 ms, Table 1), accompanied by an increase in AP amplitude and MDP, a reduced ignition period, and a reduction in cycle-to-cycle interval variability (coefficient of variance) (Figure 3A and Table 1). Among 12 initially dormant SANC, a half (six cells) began to spontaneously fire APs in response to β-AR stimulation (“responder”) (Supplementary Video 1) while the remainder (six cells) failed to do so (“non-responder”) (Figure 3B and Table 1). Steady state AP firing in the presence of β-AR stimulation in initially dormant SANC at baseline (pre-β-AR stimulation, Figure 3D) is comparable to those SANC that fired rhythmic APs at baseline of which rate accelerated in response to β-AR stimulation (Table 1 “Firing SAN β-AR stimulation” vs “Dormant SANC Responder β-AR stimulation”). Prior to β-AR stimulation, the baseline membrane potential of dormant responders did not differ from those of non-responders (Table 1). In some responder dormant SANC, membrane potential spontaneously hyperpolarized prior to the first *de novo* spontaneous AP (Figure 3C). The initial APs during β-AR stimulation were small and dysrhythmic. *Bona fide* APs subsequently began to occur within ∼20 s (Figure 3C). When β-AR stimulation was disconitinued (isoproterenol washout), the responder cells became dormant again (Figure 3E). In non-responder dormant SANC, the membrane potential did not change following β-AR stimulation (Table 1).

To determine whether β-AR stimulation-induced activation of dormant SANC was dependent on increased cAMP signaling, we repeated the same experiment with cell-permeable CPT-cAMP. CPT-cAMP recapitulated the β-AR stimulation-induced automaticity in 46% of dormant SANC studied [6/13, *p* < 0.05 vs time control (0/8, 0%), Figure 3F], indicating that the resumption of AP firing in initially dormant SANC is indeed caused by cAMP-dependent mechanisms.

### The Full Spectrum of Clock Coupling Emerges in Dormant SANC That Fire APs in Response to an Increase in cAMP

**FIGURE 4:**
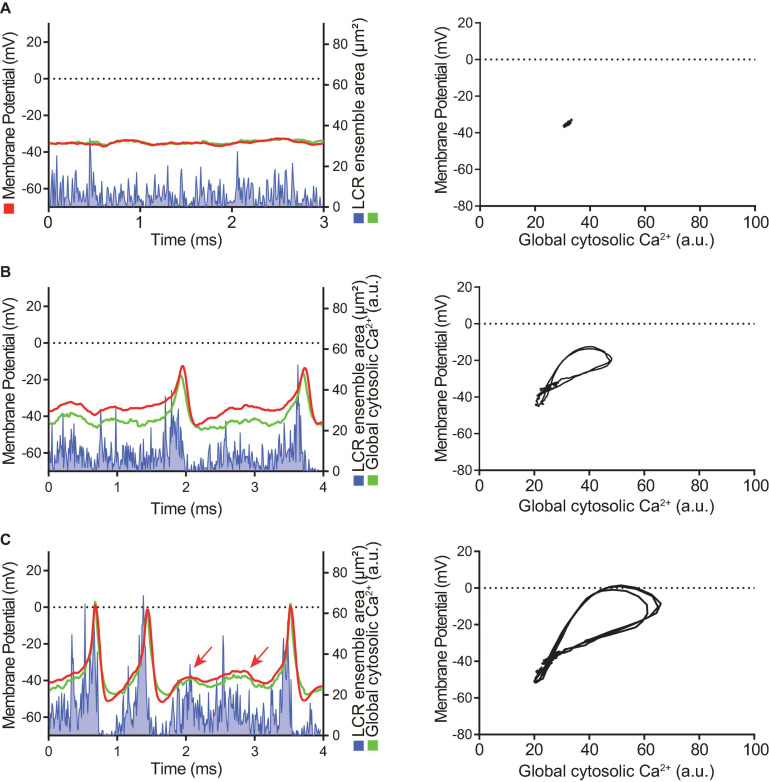
Time domain (left column) and the Ca^2+^-voltage phase-plot loops (right column) of simultaneous membrane potential (red), whole-cell Ca^2+^ transient (green) and LCR ensemble area (blue) measurement at the early stage of AP induction in response to β-AR stimulation in a SANC that was electrically dormant at baseline. Note that, to minimize both phototoxicity and bleaching of the Ca^2+^ indicator, Ca^2+^ signals could be recorded only for short time window, although membrane potential could be recorded for prolonged periods: **(A)** Baseline, **(B)** The first AP, and **(C)** Following early phase that is characterized by APs with a small amplitude and failed ignition (arrows). Blue line shows the integrated area of detected individual LCRs by our image analysis program (Maltsev et al., 2017).

**FIGURE 5:**
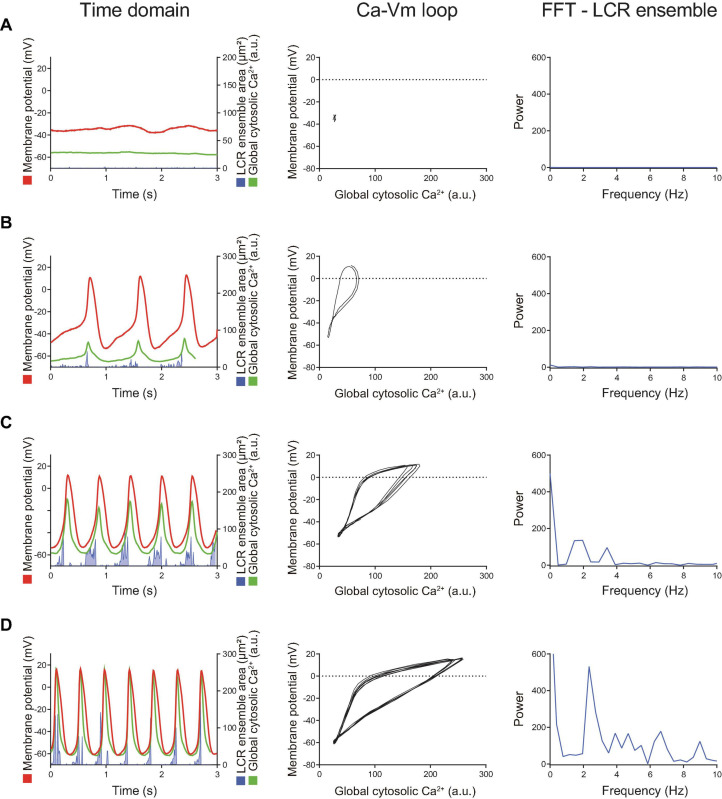
Later stage of the β-AR stimulation-induced AP induction sequence observed in other SANC that was initially dormant at baseline. Time domain (left), Ca^2 +^ -Vm loop (middle) and FFT of Ca^2+^ signals (right) during the baseline dormancy **(A)**, early stages **(B,C)**, and steady state **(D)** of AP induction in response to β-AR stimulation. The Ca-Vm loop evolves to expand as the AP cycle length shortens in response to β-AR stimulation.

Simultaneous recordings of membrane potential and 2D Ca^2+^ signals (Figures 4, 5) at critical stages of the transition from the dormancy to the spontaneous AP firing state in response to increased intracellular cAMP signaling inform on how enhanced clock coupling evolves in response to cAMP.

At baseline, while the dormant SANC failed to produce rhythmic APs or LCRs (Figure 4A), the phase-plane diagram of membrane potential vs global cytosolic Ca^2+^ signal during this sequence depicts stalled fluctuations in membrane potential and whole-cell Ca^2+^ levels in dormant SANC at baseline as a tight cluster of dots (Figure 4A right panel). In response to cAMP, the same SANC in Figure 4A generated small and slow membrane potential oscillations accompanied by a simultaneous fluctuation in whole cell Ca^2+^ and LCRs (Figure 4B left panel) progressively increasing in amplitude over time (Figure 4C left panel). Although some early small-sized APs induced small whole-cell Ca^2+^ transients, at this time the coupling degree of the two clocks failed to produce regular APs (Figure 4B left panel). As cAMP exposure time increased, AP failure still occurred but became less frequent. There was an increase in synchronization between membrane potential and Ca^2+^ toward normal size APs and Ca^2+^ transients, shown as an increase in the size of phase-plane loops over time (Figures 4B right panel, 4C right panel). The initiation of self-organizing, mutually interacting electrical and Ca^2 +^ oscillations (i.e., Ca^2+^-induced Ca^2+^ release) and its time-dependent, feed-forward augmentation following exposure to cAMP are clearly observed in another dormant SANC (Figure 5). Note two missed ignitions of full APs during Figure 4C (the 3rd and 4th “would-be firing,” indicated by arrows) were preceded by failed escalation of LCR ensemble area (Figure 4C left panel). This finding suggests the possibility that the cAMP-induced APs in initially dormant SANC are caused by emergent coupling between the M- and Ca^2 +^ -clocks. Later stages of β-AR activation demonstrated further extended the progressive increase in the “loop” size depicted in the Vm-Ca^2 +^ phase plane diagram with associated shortening of CL of cAMP-induced APs, respectively (Figures 5B–D). During steady-state AP firing (Figure 5D), the LCR ensemble is synchronized in time such that the LCR ensemble forms sharp spikes immediately before the AP upstroke. The failed interactions between Ca^2 +^ - and M-clocks that resulted in failed AP ignition observed during earlier stages (Figures 4B,C) no longer occur, and there is regular AP firing.

**FIGURE 6:**
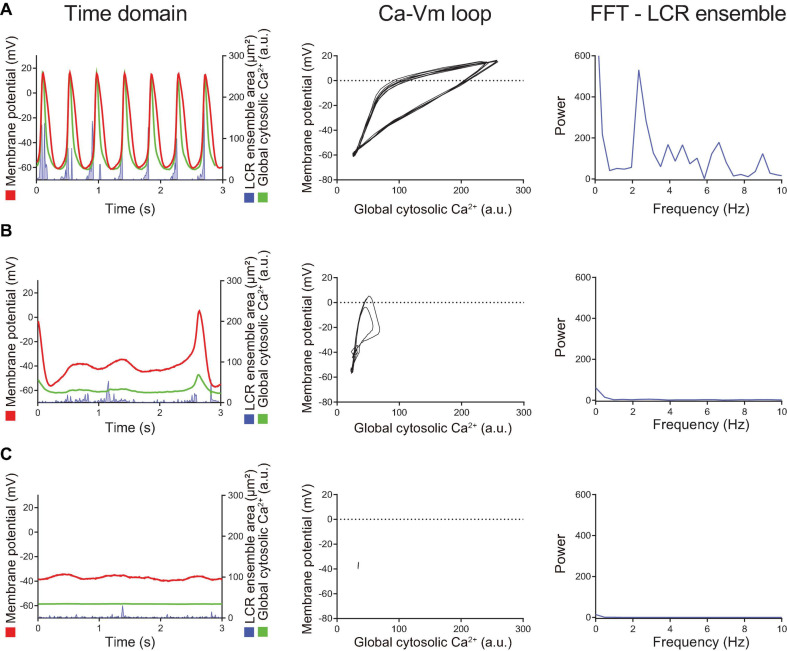
When the intracellular cAMP level decreased by washout of BARs/CPT-cAMP, the changes described in Figure 5 reversed in order. Compared to the steady state AP firing (panel **A**), the AP cycle length is prolonged and LCR ensemble is decreased (panel **B**), suggesting the disengagement of M- and Ca^2+^-clocks. Shortly after cAMP washout (the panel **B**), spontaneous AP firing ceased, and the cell returned to dormant state again (panel **C**) in which the membrane potential drifted around –40 mV, ensemble LCR signal area decreased, and the voltage-Ca^2+^ loop demonstrated only very small fluctuation around one point, rather than crating a loop.

The isoproterenol/CPT-AMP wash-out phase is characterized by symmetrical reversal of the steps described above. The SANC gradually fail to maintain regular AP firing and eventually membrane potential depolarized to ∼ −40 mV, accompanied by loss of fluctuations in membrane potential. At the same time, the whole-cell Ca^2+^ signal become markedly reduced and LCRs became small and disorganized (Figure 6). Note the similarities in the transition during “wash-out” and “wash-in” shown in Figure 6 and Figure 4, respectively.

In summary, these results indicate that clock functions and coupling are severely reduced in dormant SANC at baseline; an increase in clock coupling underlies the cAMP-dependent AP rescue in dormant SANC.

### Voltage-Dependent Ca^2+^, K, and Funny Current Increases in Dormant SANC in Response to Increasing cAMP

**FIGURE 7:**
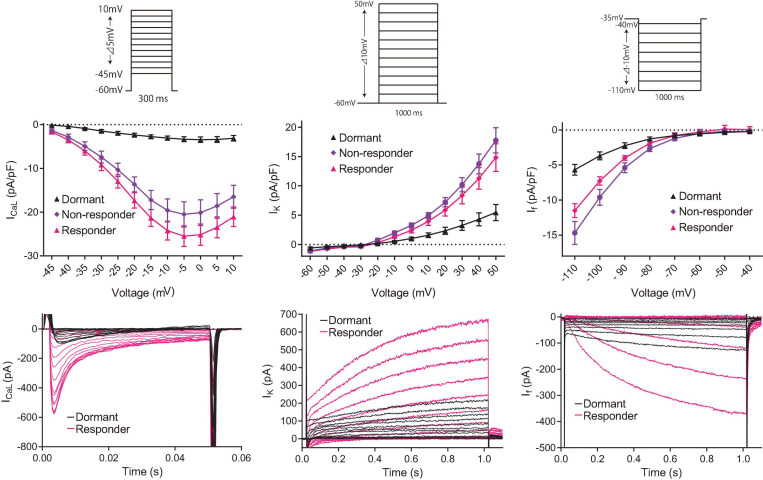
Consecutive in-series measurements of ion-current density of L-type Ca^2+^ current (left panels), K^+^ current (center panels) and funny current (right panels) from SANC revealed that M-clock function of both responder (magenta) and non-responder (purple) dormant SANC increases in response to β-AR stimulation by 100 nM isoproterenol. The voltage protocol (same to those for Figure 1C) is shown above the I-V curves. Example I–V curves for each current is shown in the bottom raw. The data shown are from different cells, one without isoproterenol (black) and one with isoproterenol (magenta).

To determine the effects of increased cAMP on M clock functions in responder and non-responder SANC, we evaluated ion current densities with voltage-clamp experiments following brief Ca^2+^ imaging (i.e., total light exposure limited to <4 s) to classify the baseline AP firing behavior of each cell and its response to isoproterenol. I_CaL_, I_K_ and I_f_ densities of initially dormant SANC increased in response to isoproterenol in both responder and non-responder cells (Figure 7), suggesting that the enhanced cAMP signaling augments M-clock function in all of these dormant cells. However, there was no difference in current density after exposure to isoproterenol between responders and non-responders, indicating that the augmentation in M-clock functions did not predict whether dormant SANC at baseline would respond to cAMP.

### Selective Independent Blocks of the Ca^2+^ and M Clock Reveal Their Different Roles in Restoration of β-AR-Induced APs in the Dormant SANC

To further characterize the relative roles of M- and Ca^2+^ clock mechanisms in the resumption of automaticity in initially dormant SANC, we compared the inducibility of APs in dormant cells by isoproterenol in the presence of CPA (0.5 μM, *n* = 11) or IVA (3 μM, *n* = 7) to that of control group (*n* = 10).

CPA, a Ca^2+^ clock inhibitor that acts by inhibiting SR Ca^2+^ ATPase (SERCA) and reducing Ca^2+^ pumping into the SR (Vinogradova et al., 2010), reversibly suppressed the LCR ensemble signal in dormant SANC, confirming that LCR signals observed in dormant SANC are SERCA-dependent. In response to subsequent β-AR stimulation, only 9% (1/11) of initially dormant SANC superfused with 0.5 μM CPA generated spontaneous APs, a significantly lower % than the control group (*p* < 0.05), in which 60% (6/10) of initially dormant SANC began to fire spontaneous APs in response to β-AR stimulation (Table 2). The baseline membrane potential of non-responder dormant SANC bathed in CPA was around −40 mV with CPA addition, and remained unchanged during and following wash out of β-AR stimulation (Table 2).

**TABLE 2:** Membrane potential characteristics of CPA/IVA-pretreated dormant SANC in response to β-AR stimulation.

	**DMSO-pretreated *n* = 10**	**CPA-pretreated *n* = 11**	**IVA-pretreated *n* = 7**
Responded cells (%)	6 (60%)	1 (9%*)	5 (71%)
**Responder**			
Dormant potential - Baseline (mV)	−41.3 ± 3.0	−46.2	−38.3 ± 0.8
CL (ms)	435 ± 17	579	1118 ± 340**
CV (%)	0.42 ± 0.04	2.3	3.3 ± 1.0**
MDP (mV)	−65.2 ± 2.0	−65.0	−58.5 ± 2.8**
AP amplitude (mV)	73.5 ± 5.2	70.6	64.7 ± 5.7
**Non-responder**			
Dormant potential - Baseline (mV)	−44.6 ± 0.6	−42.1 ± 3.2	−37.9 ± 4.8
- Isoproterenol (mV)	−42.5 ± 1.5	−42.7 ± 3.8	−42.3 ± 2.1

*p < 0.05 vs DMSO-pretreated group by Fisher’s exact test. **p < 0.05 vs DMSO-pretreated group by unpaired *t*-test. CV stands for coefficient of variance (calculated as standard deviation divided by mean).

In contrast to CPA, pretreatment of SANC with selective if inhibitor ivabradine (3 μM) did not affect the success rate (71%, 5/7) of β-AR stimulation in restoring AP firing in initially dormant SANC (Table 2). However, the AP CL of the IVA-pretreated responder dormant SANC during β-AR stimulation was longer and more irregular, and the MDP was relatively depolarized, compared to control responder SANC during β-AR stimulation (Table 2).

### Phosphorylation of Coupled-Clock Proteins Is Reduced in Dormant SANC at Baseline

PLB is a protein that modulates the Ca^2+^ kinetics of the Ca^2+^ clock by controlling the pumping function of SERCA. We analyzed the phosphorylation of PLB in Serine 16, the main site of this regulatory protein activated by cAMP-dependent protein kinase A (PKA), by quantifying the phosphorylated-PLB/total PLB ratio (pS16-PLB/total PLB ratio) following measurement of Ca^2 +^ dynamics in the same cells. Phosphorylation of Serine 16 was reduced by sixfold in dormant SANC vs SANC firing spontaneous APs in normal Tyrode under basal conditions (Figures 2A,B).

**FIGURE 8:**
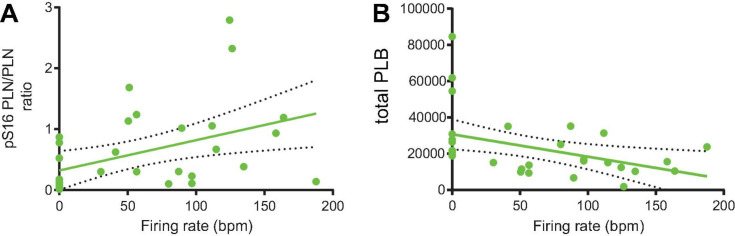
The phosphorylated-PLB/total PLB ratios **(A)** and total PLB number **(B)** and were positively (*n* = 32; *Y* = 0.004982X + 0.3209, *p* = 0.0132; *R*^2^ = 0.188) and negatively (*n* = 31; *Y* = –123.5X + 30723, *p* = 0.0161; *R*^2^ = 0.184) associated with the firing rate, respectively.

Next, we perfused a subgroup of SANC that fired APs at baseline with normal Tyrode supplemented with isoproterenol 100 nM to prove that an increase in AP firing rate in these cells is associated with an increased PLB phosphorylation. In response to β-AR stimulation, the phosphorylation level of PLB increased by an additional twofold over that level observed in the group of cells firing APs at baseline in the absence of β-AR stimulation (Figures 2A,C). We further found that in SANC we studied, including those dormant cells (firing rate = 0) the phosphorylated-PLB/total PLB ratios were positively correlated with AP firing rate (Figure 8A). In contrast, when the absolute number of PLB counts were plotted as a function of AP firing rate, they were negatively correlated (Figure 8B).

## Discussion

Our results demonstrate that augmentation of cAMP-PKA signaling is capable of restoring automaticity of dormant cells via enhancements in both M- and Ca^2+^-clock functions and their coupling, including increases of key membrane currents I_CaL_, I_K_, and I_f_ and phosphorylation of the critical SR Ca^2+^-clock protein PLB.

### Role of cAMP Signaling in SANC Normal Baseline Automaticity

As we noted in Introduction, in SANC that fire APs at baseline, intracellular cAMP-PKA signaling is maintained around the middle of the range of possible signaling levels (Vinogradova et al., 2008), resulting in a moderate degree of coupling between the roughly periodic SR Ca^2+^-clock and the membrane-bound, voltage- and time-dependent M clock that together drive basal AP firing. Thus, direct cAMP binding to certain clock proteins (e.g. HCN4) and cAMP mediated-PKA dependent phosphorylation of other clock proteins (e.g., PLB, RyR, K^+^-channels and L-type Ca^2+^ channels) is what drives basal SANC automaticity (Lakatta et al., 2010). This spontaneous AP firing and its attendant Ca^2+^ influx maintain cytoplasmic Ca^2+^ at a level sufficient to support the mean basal AP firing rate and rhythm by further activating Ca^2+^-dependent adenylyl cyclases and CaMKII. To keep this robust feed-forward system of phosphorylation in check, counteracting phosphodiesterases and protein phosphatases are simultaneously activated (Lakatta et al., 2010; Tsutsui et al., 2016). A change in the degree of the clock coupling is accompanied by changes in the mean AP firing CL.

### Enhanced Clock Coupling in Response to Increased cAMP-PKA Signaling

To accelerate the mean AP firing rate of single isolated SANC, β-AR stimulation-induced augmentation of clock phosphorylation more precisely synchronizes LCRs in time. This augmented LCR ensemble Ca^2+^ signal produces a larger I_NCX_ earlier in diastole (Maltsev and Lakatta, 2010). This earlier and greater Ca^2+^-NCX interaction during β-AR stimulation occurs in addition to the cAMP-mediated augmentation of I_f_ and I_K_ (Lyashkov et al., 2018). The β-AR-induced reduction in mean AP CL is accompanied by a reduction in the inter-AP interval variability (Monfredi et al., 2014).

Dormant SANC, studied here, however, appear to lack of effective functions M- and Ca^2 +^ - clocks and their interactions, producing only small and disorganized LCRs (Figures 1C, 5A). M-clock functions in dormant SANC (I_CaL_, I_f_, and I_K_ densities and I–V relations) were reduced compared to those in SANC firing spontaneous AP at baseline (Figure 1B). We interpret our results to indicate that in the dormant state, a disorganized Ca^2+^ signal occurs but no APs are ignited, this is a manifestation of suppression of both the M- and Ca^2+^- clocks. The relatively depolarized dormant SANC membrane potential of ∼-38 mV will undoubtedly hamper the voltage-dependent mechanisms within the M-clock.

We observed that, as in SANC spontaneously firing APs at baseline (reviewed in Irisawa et al., 1993 and Mangoni and Nargeot, 2008), β-AR stimulation in initially dormant SANC increased three major inward and outward currents: Ca^2+^, K^+^ and funny currents. Therefore, it is reasonable to speculate that an increase in the M-clock function mediated by augmentation of the currents that we studied plays an important role in the “responder” SANC. With β-AR stimulation, density of ion currents increases, and this is the result of a phosphorylation of the channel and/or a direct action of cAMP on the channel. However, a striking finding was that the effect of β-AR stimulation on these current densities did not differ between responders and non-responders (Figure 7), that is this effect of β-AR stimulation is not *sufficient* to guarantee transformation from dormancy to automaticity. This indicates that there must be additional mechanisms for automaticity to be restored in dormant SANC.

Ca^2+^ clock function is also decreased in dormant SANC, compared to SANC that fire spontaneous APs at baseline. The absence of regular AP-induced Ca^2+^ influx leads to a reduction in cytosolic Ca^2+^ level (Figure 6) (van Borren et al., 2010; Tsutsui et al., 2018). This low Ca^2+^ level impacts on the robustness and rhythmicity of spontaneous LCRs. However, baseline SR Ca^2+^ content of guinea pig dormant SANC is comparable to that of firing SANC, and it neither increases nor decreases in response to β-AR stimulation (Kim et al., 2018). The absence of increased SR Ca^2+^ load in a dormant SANC demonstrated in our previous research (Kim et al., 2018) may be due to low cytosolic Ca^2+^ that diminishes the ratio of phosphorylated PLB to total PLB suppressing SR function (Sirenko et al., 2012). The metabolic state including ATP production would also be expected to be reduced during dormancy compared to SANC that fire APs at baseline. In such a state, Ca^2+^-activated AC and PKA and CaMKII signaling, which is activated in basal state in SANC, would also be expected to decrease, causing a reduction in a wide range of cell functions within SANC. Important evidence for critical roles of basal PKA and CaMKII signaling in transition to dormancy is that a selective inhibition of either signaling (AIP or PKI) results in cell dormancy with a membrane potential fluctuating at about −30 mV (Vinogradova et al., 2000; Vinogradova et al., 2006). The important role of Ca^2+^ for cell dormancy have been demonstrated in previous studies: Inhibition of Ca^2+^ cycling (ryanodine or BAPTA-AM) facilitated vagally induced SANC dormancy (van Borren et al., 2010). Our previous studies in permeabilized SANC showed that LCRs are not random openings of RyR2 and they are different from Ca^2+^ sparks in ventricular myocytes (Sirenko et al., 2012). LCRs exhibit a larger spatial extent (appeared manly as locally propagating wavelets) and their occurrence is partially periodic, i.e., LCRs behave as a set of heterogeneous local Ca^2+^ oscillators (or “clocks”), whereas occurrence of Ca^2+^ sparks in ventricular myocytes at the basal state is mainly random. In this regard, it has previously been reported that freshly isolated single permeabilized ventricular myocytes, exhibiting random Ca^2+^ sparks in a physiologic free (Ca^2+^), manifest a marked self-organization of Ca^2+^ sparks to produce robust and roughly periodic LCRs when PLB becomes highly phosphorylated during PDE and phosphatase inhibition (Sirenko et al., 2012, 2014).

Our results demonstrate that, as a marker of SANC-wide coupled clock protein PLB, phosphorylation at Serine 16 is markedly reduced in dormant SANC vs those that fire spontaneous APs in dormant SANC. Furthermore, the phosphorylated-PLB/total PLB ratios were positively correlated with AP firing rate (Figure 8A), whereas the absolute numbers of PLB were negatively correlated (Figure 8B), indicating that SR Ca^2+^ pumping in dormant cells is likely to be suppressed by two mechanisms: low PLB phosphorylation and high PLB expression. Insufficient SR Ca^2+^ pumping, in turn, could inhibit Ca^2+^ clock operation and contribute to cell dormancy. Increased cAMP-dependent phosphorylation of PLB enables these dormant cells to fire APs. In response to β-AR stimulation, both the baseline function of Ca^2+^ clock (Figures 4, 5) and M clock (Figure 7) in dormant SANC becomes enhanced. As a result, the Ca^2+^-membrane potential phase plane diagram (Figures 4–6), a visual representation of the Ca^2+^-Na^+^ electro-chemical gradient oscillation that underlies an AP cycle, evolves from a tight cluster of dots at baseline (Figures 4A, 5 top) to the narrow, early transition stage, to the steady state, in which the membrane potential fluctuation within the same range is accompanied by larger intracellular Ca^2+^ oscillations (Figure 5). This suggests that an increase in clock coupling emerges upon the cAMP-dependent activation of dormant SANC; and that clock coupling continues to increase in the context of the time-dependent increase in intracellular Ca^2+^ and increase in cAMP-PKA signaling that enhances function of proteins of both clocks. Note that the low *R*^2^ values between the AP firing rate and p-/total PLB ratio (Figure 8) indicates that the impact of factors that determine AP firing rate varies from cell to cell.

### Specific Biophysical Mechanisms

With respect to specific biophysical mechanisms, the transition from dormancy to AP firing may be explained by impact of the enhancement of cAMP-PKA signaling on the ignition process, i.e., I_f_, LCRs and feed-forward interactions of Ca^2+^ and M clock, including LCRs, NCX, and I_CaL_ (Lyashkov et al., 2018). Following the ignition theory, increased LCRs and I_NCX_ depolarize cell membrane activating low voltage-activated Ca channels (Ca_v1.3_ and Ca_v3.1_) that generate both respective diastolic Ca currents (I_CaL_ and I_CaT_) and attendant Ca^2+^ influx to activate more LCRs via Ca^2+^-induced-Ca^2+^-release (Chen et al., 2009; Torrente et al., 2016). The additional LCRs, in turn, generate more I_NCX_ and membrane depolarization, forming an explosive feed-forward loop to insure robust ignition of a new pacemaker cycle. Our previous study of dormant cells (Kim et al., 2018) featured a numerical model of this transition from dormancy to AP firing. Numerical simulations of this model illustrated that the diastolic I_NCX_ amplitude substantially increases from a steady level of about −4 pA in dormant cells to oscillations from −9 to −15 pA in firing cells (Supplementary Figures 1, 2).

The results of ivabradine and CPA experiments (Table 2) suggest that enhanced function of the Ca^2+^-clock is critical for the majority of responder dormant SANCs to initiate the *bona fide* APs in response to β-AR stimulation, while I_f_ current, in contrast, appears to have a role in stabilization of the β-AR stimulation-induced *de novo* APs among responder dormant SANC. This interpretation is supported by previous studies (Vinogradova et al., 2006) that demonstrated the obligatory role of basal PKA activation for normal SANC automaticity (that is opposite to dormancy): specific inhibition of PKA-dependent phosphorylation by PKI results in AP firing failure, i.e., cell dormancy. Thus, PKA-insensitive I_f_ directly regulated by cAMP (DiFrancesco and Tortora, 1991) is expected to remain unchanged under these conditions of selective PKA inhibitions, but it is not sufficient to prevent dormancy.

One important specific question is about why dormant cells have a depolarized membrane potential fluctuating near −38 mV (Figure 1B) and what causes membrane hyperpolarization during their transition to AP firing (Figure 3B). Previous studies showed that a reduction in the electrochemical gradient by decreasing extracellular Na^+^ or increasing extracellular K^+^, pharmacological blockade of I_Kr_ (Verheijck et al., 1995) or I_CaL_ (Verheijck et al., 1999), or a brief, subthreshold depolarizing or hyperpolarizing pulse (Jalife and Antzelevitch, 1979) induces dormancy in SANC that otherwise spontaneously fire APs at either the single cell or tissue-ball level. Dormant SANC can be reactivated by reverting the altered extracellular ion composition (Noma and Irisawa, 1975a), adrenergic agonists (Opthof et al., 1987), or subthreshold pacing to cardiac neurons that induce I_KACh_-driven hyperpolarization to an adjacent dormant SANC (Goto et al., 1983). The predesposition to dormancy can be also linked to substantial functional heterogeneity of key ion current densities, i.e., the M clock side of the system (Honjo et al., 1996; Monfredi et al., 2018).

The existence of “pseudo resting” potential of −30 to −40 mV, when voltage-gated channels are not active, has been thoroughly studied and discussed by Capel and Terrar (2015a, b) who noted that upon cessation of rhythmic AP firing (by multiple different reasons) many SANC fluctuate around a membrane potential in the region of −35 mV. A similar “resting potential” of −38 mV was also observed in rabbit SA node by Noma and Irisawa (1975b). It was suggested that this relatively depolarized level of membrane potential is determined by a balance of numerous ion currents of different nature (Capel and Terrar, 2015b), and I_NCX_ seems to also critically contribute to this balance (Sanders et al., 2006). The relatively depolarized membrane potential of −38 mV in dormant cells could be also explained by the absence of I_K1_ current in SANC combined with a lower amplitude of voltage-activated K^+^ currents (in the absence of their activation by APs). We tested this idea via numerical model simulations (Supplementary Figure 3) but found that in a dormant cell I_Kr_ remains relatively high, near 15 pA (blue line), i.e., larger than I_NCX_ amplitude of about 4 pA (Supplementary Figure 2), indicating that a lower K^+^ current amplitude is hardly the only reason of the depolarized potential observed in dormant cells.

Future studies should further address the mechanisms that drive the initial hyperpolarization in responding dormant cells. One possible mechanism is via electrogenic transporters (i.e., not ion channels) such as Na^+^/K^+^ pump. Hyperpolarization can be also driven by Ca^2+^-activated K^+^ channels [review (Clements et al., 2015)]. All three “small K” isoforms (SK1, SK2, and SK3) were identified in mouse SAN (Torrente et al., 2017) and inhibition of SK channels with apamin prolonged APs in isolated SAN cells, slowed diastolic depolarization, and reduced pacemaker rate in isolated SAN cells and intact SAN tissue (Torrente et al., 2017). Large-conductance Ca^2+^- and voltage-activated K^+^ channels (BK channels or maxi-K^+^ channels) also seem to play a prominent role in pacemaker function (Imlach et al., 2010; Lai et al., 2014).

### Are Dormant SANC Functional Members of a Heterogeneous Pacemaker Cell Community in Intact SAN Tissue?

It has been reported that most of the SANC enzymatically isolated from guinea pig SAN do not beat spontaneously (Toyoda et al., 2018). This has traditionally been interpreted as a result of damage from the cell isolation process. However, one might consider that the AP firing behavior of a single isolated SANC would not necessarily be identical to that of intact SAN tissue. Rather than representing damaged SANC, recent research (Kim et al., 2018; Tsutsui et al., 2018) and the present study demonstrate that almost half of SANC that do not beat at baseline “wake up” in response to increased cAMP-PKA signaling and began to fire spontaneous APs at a rate that does not differ from that of isolated SANC that fire AP at baseline; and the cells return back to dormant state when the stimulus is removed. While dormant cells studied here would likely behave differently within a cellular network of SAN, the possibility remains that (i) such dormant SANC may exist in intact SAN tissue and can dynamically change the ensemble AP firing behavior, or (ii) AP firing cells become the dominant cells in certain prevailing physiological conditions that may not favor the phenotype of the SANC that beat spontaneously at baseline after isolation.

Previous studies have a bearing on this issue. Studying surgically isolated small SAN tissue preparations, Opthof et al. (1987) observed that, while the majority of small SAN tissues demonstrated spontaneous AP firing at different frequencies, some of the small SAN tissues dissected from the septal side of the SAN were electrically dormant. Some SANC within SAN tissue are electrically quiescent during normal heart function (Noma and Irisawa, 1975a,b; Goto et al., 1983; Opthof et al., 1987; Toyoda et al., 2018). This is believed to reflect a degree of functional redundancy – the SAN of cats contains about 2000 primary pacemaker cells, but can function normally with less than 500 cells (Opthof et al., 1986). A physiological implication of this phenomenon is that not all SANC contribute to all beats; this may be to optimize energy consumption at times when the participation of all SANC within SAN tissue is not required, e.g., in the basal state. Vagal nerve stimulation modulates SAN rate and rhythm and can lead to marked sinus bradycardia or even arrest (van Borren et al., 2010). Non-firing SANC may be able to serve as subsidiary pacemakers by responding differently to external stimuli, thereby resulting in a shift of the “leading pacemaker site” within the SAN (Lang et al., 2011). However, the actual physiological role of non-firing SANC *in vivo* remains unclear.

The function of dormant cells will be better realized within the context of evolution of our understanding of SAN tissue operation. About 40 years ago SANC automaticity has been conceptualized as initiated by small group of “strong” cells (a dictator model) with a concentric propagation from the initiation site toward atria (Sano et al., 1978; Bleeker et al., 1980). Then an idea of mutual entrainment of coupled oscillators (Winfree, 1967) was applied to the coordinated firing of the entire population of SAN cells (Jalife, 1984; Michaels et al., 1987): individual SAN cells that are loosely connected through low resistance junctions generate spontaneous excitations that differ in phase, mutually entrain each other to fire with a common period. Recently, by utilizing a newly developed whole-tissue Ca^2+^ imaging apparatus, we have discovered an extensive, reticular HCN4+/Cx43-cell meshwork across the posterior aspect of murine right atria along the crista terminalis that generates spontaneous *regular* electrical impulses (Bychkov et al., 2020). Within the network, however, we have observed highly heterogeneous AP firing behavior. Some SANC within this network discharge spontaneous APs regularly, while others had dysrhythmic AP firing, and even some subpopulations remain completely dormant. The majority of these SANC had LCRs along with the intricate and complex cell-to-cell heterogeneous Ca^2+^ signals. That study proposed a novel, microscopic signaling paradigm of SAN operation in which synchronized APs emerge from heterogeneous subcellular subthreshold Ca^2+^ signals, resembling multiscale complex processes of impulse generation within clusters of neurons in neuronal networks. Another recent study (Fenske et al., 2020) also detected non-firing cells in mouse SAN and provided evidence that a tonic and mutual interaction process (tonic entrainment) between firing and non-firing cells slows down the overall rhythm of the SAN.

## Conclusion

Our results demonstrate that cAMP-signaling awakes AP firing of dormant SANC via emergence of a coupled-clock system, i.e., the emergence of self-organized roughly periodic Ca^2+^ clock and concurrent oscillations of membrane potential that informs on an increase in clock synchronization in response to β-AR stimulation. The results of the present study raise, but do not prove, the possibility of the existence of dormant SANC *in vivo*; but if this were the case, dynamic recruitment of these cells *in vivo* would likely depend on cAMP-PKA signaling. In other words, some cells that are embedded in SAN tissue, but do not generate APs all the time (Bychkov et al., 2020; Fenske et al., 2020), may activate to fire APs conditionally, namely in the presence of β-AR stimulation *in vivo*. Other cells may deactivate upon cholinergic receptor stimulation (Goto et al., 1983; Opthof et al., 1987; Fenske et al., 2020).

### Limitations and Future Directions

Although substantial evidence has recently emerged to indicate the presence of non-firing cells in intact tissue, we must emphasize that dormant cells we study in isolation are not necessarily the same dormant cells that have been observed in the intact tissue. More experimental and numerical studies are needed to clarify the role of non-firing cells in SA node and biochemical (cAMP, PKA, and CaMKII signaling) and biophysical mechanisms of dormant cell transition to AP firing, including initial hyperpolarization. Multi-cellular and multiscale numerical modeling would include dormant cells and test several new hypotheses of SAN operational paradigm that have been proposed but not validated yet, including the ideas of subthreshold signal summation among neighboring cells (Bychkov et al., 2020), stochastic resonance (Clancy and Santana, 2020), percolating criticality (Weiss and Qu, 2020), and cell silencing at lower rates (Fenske et al., 2020).

## Data Availability Statement

The raw data supporting the conclusions of this article will be made available by the authors, without undue reservation.

## Ethics Statement

The animal study was reviewed and approved by the Animal Care and Use Committee of the National Institutes of Health.

## Author Contributions

KT: perform the experiments, and drafting of the manuscript and critically revising it for important intellectual content, analysis, and interpretation of the data. MF, AY, AW, DY, MK, and BZ: perform the experiments, and critically revising the manuscript for important intellectual content, analysis, and interpretation of the data. RB and OM: analysis and interpretation of the data. VM: project planning, analysis and interpretation of the data, numerical model simulations, and drafting of the manuscript and revising it critically for important intellectual content. EL: project planning, conception and design of the experiments, and drafting of the manuscript and revising it critically for important intellectual content. All authors contributed to the article and approved the submitted version.

## Conflict of Interest

The authors declare that the research was conducted in the absence of any commercial or financial relationships that could be construed as a potential conflict of interest.

## Supplementary Material

Supplementary Movie 1 Simultaneous measurement of Ca^2 +^ and voltage signals from an initially dormant SANC that began to fire in response to CPT-cAMP. At the baseline, although membrane potential hovers around −40 mV, LCRs are present. During the transitions, the same cell began to fire spontaneous AP (for detailed description, see text and Figures 5, 6). Following the CPT-cAMP washout, the SANC ceases to fire spontaneous AP, turned dormant again.
