# Supplementary material for: cAMP-Dependent Signaling Restores AP Firing in Dormant SA Node Cells via Enhancement of Surface Membrane Currents and Calcium Coupling
Source: Front Physiol. 2021 Apr 9;12:596832. doi: 10.3389/fphys.2021.596832 (PMC8063038; doi:10.3389/fphys.2021.596832)
Supplement: Supplementary Movie 1 — Simultaneous measurement of Ca2 + and voltage signals from an initially dormant SANC that began to fire in response to CPT-cAMP. At the baseline, although membrane potential hovers around −40 mV, LCRs are present. During the transitions, the same cell began to fire spontaneous AP (for detailed description, see text and Figures 5, 6). Following the CPT-cAMP washout, the SANC ceases to fire spontaneous AP, turned dormant again. [file Data_Sheet_1.PDF]

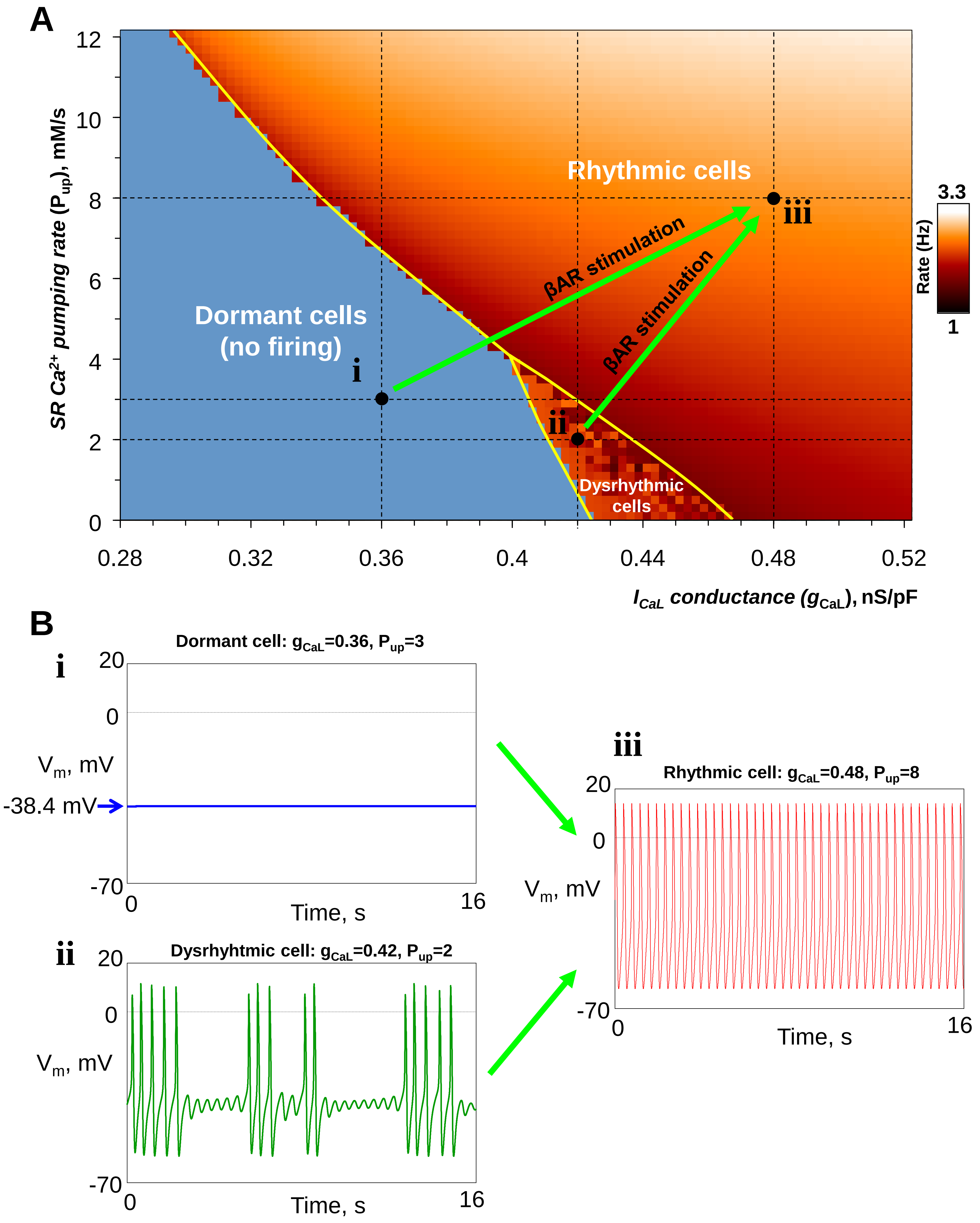

Figure S1. Parametric sensitivity analysis of a coupled-clock model of SANC function reveals the existence of dormant, dysrhythmic, and rhythmic cells and also offers an explanation of their rhythmic firing in the presence of βAR stimulation. **A**, color-coded AP firing rate (red shades) generated by 5,917 numerical models, based on 2009 Maltsev-Lakatta model that features 12 ion currents of a "membrane clock" and a "Ca clock". The 2d sensitivity analysis was performed by varying maximum SR Ca pumping rate (P_{up}) and maximum I_{CaL} conductance (g_{CaL}). All other parameters were assigned to their original values and kept unchanged. Modified from Maltsev & Lakatta, *Am J Physiol Heart Circ Physiol* 2009;296:H594-H615 and from Kim et al. *Cell Calcium* 2018;74:168-79. **B**, membrane potential traces simulated by typical models of dormant, dysrhythmic, and rhythmic cells indicated in panel A by small black circles. The dormant cell model shows a resting potential of -38.4 mV (blue arrow in sub-panel **i**). As g_{CaL} and P_{up} reportedly increase in the presence of βAR stimulation (green arrows), the cells generate rhythmic APs.

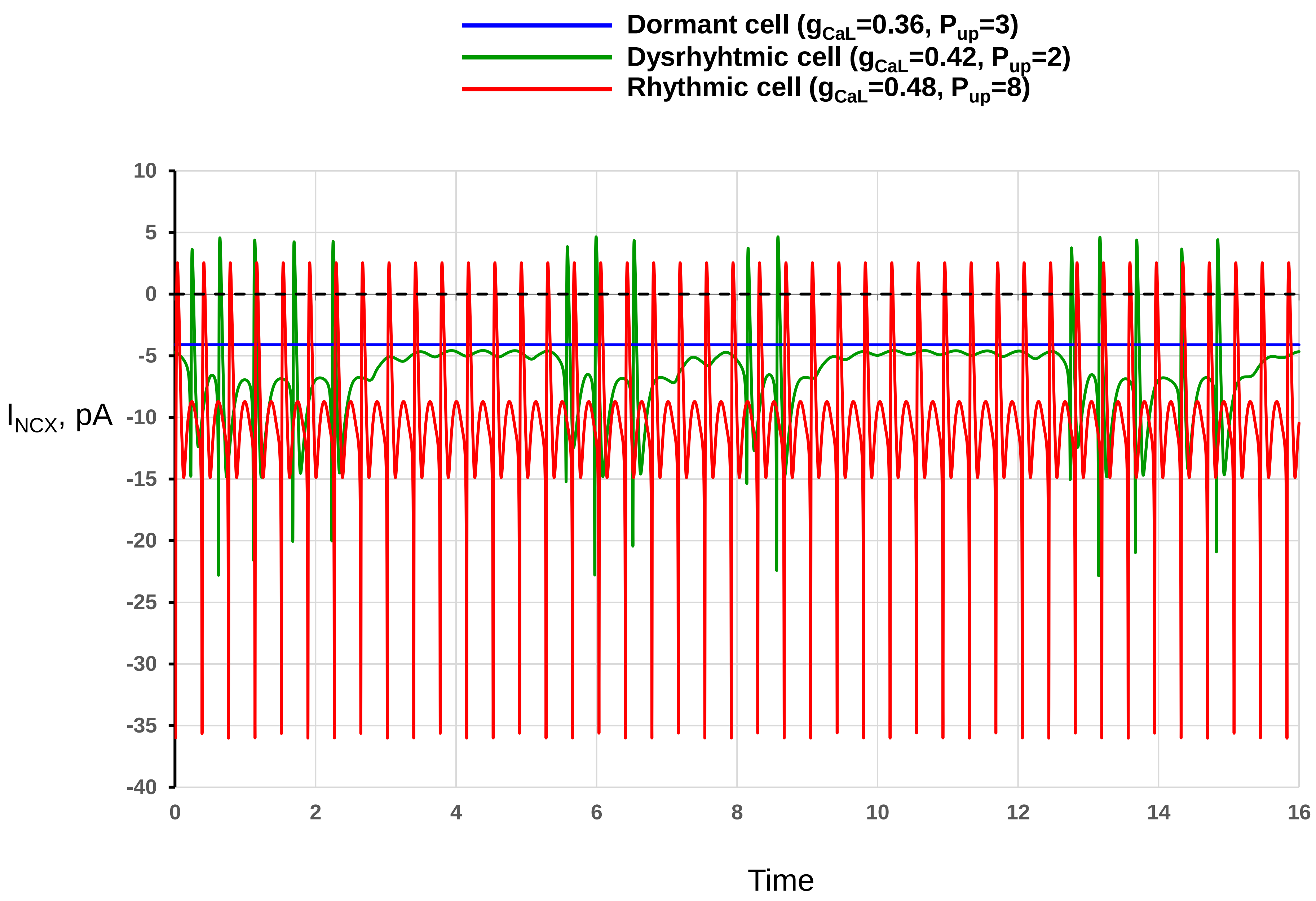

Figure S2. Clocks coupling in silico: Numerical simulations of Na^+/Ca^{2+} exchanger current (I_{NCX}) in typical models of a dormant cell, dysrhythmic cell and rhythmic cell in which I_{NCX} couples Ca^{2+} clock and membrane clock.
Numerical simulations were performed using a coupled-clock pacemaker cell model (Maltsev and Lakatta. *Am J Physiol Heart Circ Physiol* 2009; 296:H594-H615). Using the results of our broad parametric sensitivity analysis shown in Figure S1, the values of g_{CaL} and P_{up} were chosen (shown in parenthesis) to represent typical experimentally measured behaviors of dormant, dysrhythmic, and rhythmic cells. Dashed line shows zero current level. Modified from Kim et al. *Cell Calcium* 2018;74:168-79.

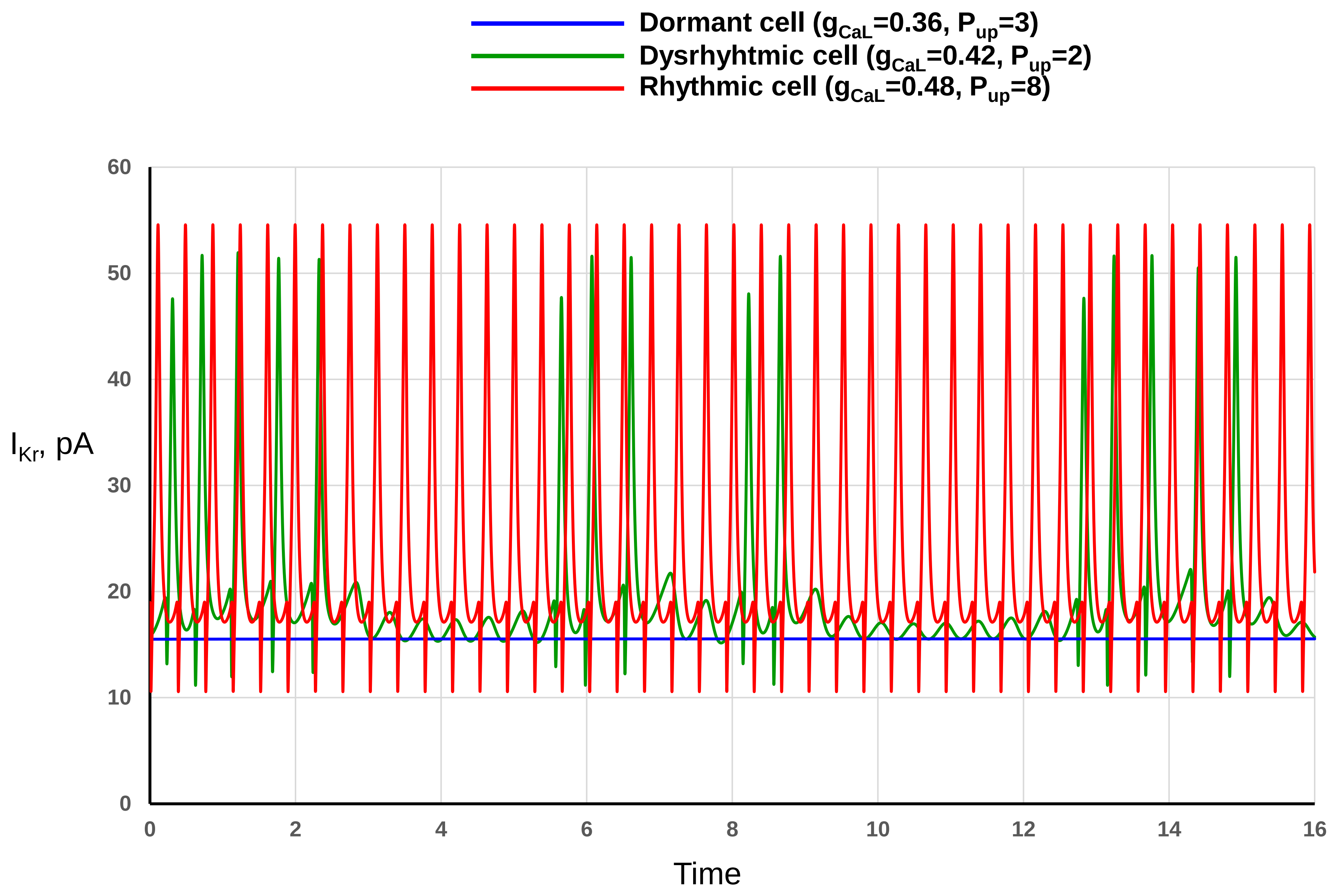

Figure S3. Numerical model simulations of delayed rectifier K^+ current (I_{Kr}) in typical models of a dormant cell, dysrhythmic cell and rhythmic cell. Numerical simulations were performed using a coupled-clock pacemaker cell model (Maltsev and Lakatta. Am J Physiol Heart Circ Physiol 2009; 296:H594-H615).
